# Global analysis of transcriptionally engaged yeast RNA polymerase III reveals extended tRNA transcripts

**DOI:** 10.1101/gr.205492.116

**Published:** 2016-07

**Authors:** Tomasz W. Turowski, Ewa Leśniewska, Clementine Delan-Forino, Camille Sayou, Magdalena Boguta, David Tollervey

**Affiliations:** 1 Wellcome Trust Centre for Cell Biology, University of Edinburgh, Edinburgh EH9 3JR, Scotland;; 2 Institute of Biotechnology, Faculty of Chemistry, Warsaw University of Technology, 00-664 Warsaw, Poland;; 3 Institute of Biochemistry and Biophysics, Polish Academy of Sciences, 02-106 Warsaw, Poland

## Abstract

RNA polymerase III (RNAPIII) synthesizes a range of highly abundant small stable RNAs, principally pre-tRNAs. Here we report the genome-wide analysis of nascent transcripts attached to RNAPIII under permissive and restrictive growth conditions. This revealed strikingly uneven polymerase distributions across transcription units, generally with a predominant 5′ peak. This peak was higher for more heavily transcribed genes, suggesting that initiation site clearance is rate-limiting during RNAPIII transcription. Down-regulation of RNAPIII transcription under stress conditions was found to be uneven; a subset of tRNA genes showed low response to nutrient shift or loss of the major transcription regulator Maf1, suggesting potential “housekeeping” roles. Many tRNA genes were found to generate long, 3′-extended forms due to read-through of the canonical poly(U) terminators. The degree of read-through was anti-correlated with the density of U-residues in the nascent tRNA, and multiple, functional terminators can be located far downstream. The steady-state levels of 3′-extended pre-tRNA transcripts are low, apparently due to targeting by the nuclear surveillance machinery, especially the RNA binding protein Nab2, cofactors for the nuclear exosome, and the 5′-exonuclease Rat1.

Transcription of nuclear DNA in eukaryotes is carried out by at least three different RNA polymerases. RNA polymerase III (RNAPIII) is specialized for high-level synthesis of small noncoding RNAs. The most abundant products of RNAPIII-dependent transcription are the 5S rRNA and the many pre-tRNA species. In addition, RNAPIII synthesizes numerous, less abundant small RNAs that are involved in diverse cellular processes, including protein translocation and the processing of pre-rRNA and pre-tRNAs (Dieci et al. 2013).

The nuclear genome of *Saccharomyces cerevisiae* contains 275 actively transcribed tRNA genes (including a tRNA of undetermined specificity [tX(XXX)D]). These are grouped into 20 isotypes, each charged with a single amino acid, which are subdivided into 41 isoacceptors that each recognize the same anticodon sequence(s) (Hani and Feldmann 1998; Chan and Lowe 2009). The reported lengths of the primary transcripts vary between 72 and 133 nucleotides (nt), and ∼25% of pre-tRNAs include introns. Primary pre-tRNA transcripts undergo 5′ and 3′ maturation and intron excision to generate the mature tRNAs.

In tRNA genes, the transcription machinery recognizes conserved promoter elements, termed box A and box B, which are located within the transcribed region and form a bipartite binding site for the six-subunit basal transcription factor TFIIIC (Acker et al. 2013). Box A starts at position +8 of the mature tRNA, and the transcription start site is most frequently located 18–20 nt upstream (Dieci et al. 2013). Within yeast tRNA genes, boxes A and B are localized 31–93 nt apart and correspond to the universally conserved D- and T-loops in the tRNA structure. Internally located A and B boxes are also the main *cis*-acting control elements for most other RNAPIII transcription units.

Among nuclear RNA polymerases, RNAPIII has the most direct-acting termination signals, which consist of a tract of A residues on the template DNA strand; minimum lengths are reported to be A_4_ for human and A_5_ or A_6_ for yeast (Braglia et al. 2005; Iben and Maraia 2012; Arimbasseri et al. 2013). The distinctive presence of a 3′ poly(U) tract in the transcript apparently makes prediction of the 3′-ends of RNAPIII transcription units relatively easy (Dieci et al. 2013). However, recent data indicate that the RNAPIII termination signals are present on both the template and nontemplate DNA strands. Interactions between oligo(dA) in the template strand and oligo(U) in the nascent transcript acts as a principal destabilizing signal, while the nontemplate strand oligo(dT) tract promotes polymerase pausing, formation of the pretermination complex, and transcript release (Arimbasseri and Maraia 2015).

RNAPIII transcription rates are regulated by the highly conserved repressor Maf1 and tightly coupled to environmental conditions. Maf1 binds the RNAPIII subcomplex C82/34/31, blocking recruitment of RNAPIII to a preinitiation complex formed by the initiation factor TFIIIB and the promoter DNA (Cabart et al. 2008; Vannini et al. 2010). Maf1 was originally identified in yeast but is conserved in all eukaryotes (Pluta et al. 2001). Notably, Maf1 represses RNAPIII when cells are transferred from glucose-containing medium to nonfermentable carbon sources, such as glycerol, particularly at elevated temperature (37°C) (Ciesla et al. 2007).

RNAPIII transcripts are targets for active surveillance pathways (for review, see Wichtowska et al. 2013). Pre-tRNAs with defects in modification or folding are degraded both by the 5′-exonuclease Rat1 and by the exosome nuclease complex (Chernyakov et al. 2008; Gudipati et al. 2012; Schneider et al. 2012). Targeting of pre-tRNAs to the exosome involves cofactors, including the Nrd1-Nab3 heterodimer and the Trf-Air-Mtr4 polyadenylation (TRAMP) complex (Kadaba et al. 2006; Jamonnak et al. 2011; Wlotzka et al. 2011). Within TRAMP, Pap2 (commonly known as Trf4) or Trf5 oligoadenylate targets RNAs, while the DExH box RNA helicase Mtr4 loads the RNA into the exosome for degradation (Jia et al. 2011; Falk et al. 2014). Catalytic activity in the nuclear exosome is provided by two subunits: Dis3 (also known as Rrp44), which shows both endonuclease and 3′-exonuclease activity, and the 3′-exonuclease Rrp6 (for review, see Chlebowski et al. 2013). Rrp6 interacts with the RNA binding protein Nab2 (Schmid et al. 2012), which was recently reported to bind both RNAPIII transcripts and the polymerase (Reuter et al. 2015). Analyses of strains defective in both Dis3 and Rrp6 activity indicate that a substantial fraction of pre-tRNA transcripts are normally degraded by the exosome (Gudipati et al. 2012; Schneider et al. 2012).

The putative total RNAPIII transcriptome was initially identified in *S. cerevisiae* by genome-wide analyses using chromatin immunoprecipitation (ChIP) (Harismendy et al. 2003; Roberts et al. 2003; Moqtaderi and Struhl 2004). However, ChIP analysis is not strand specific and generally has limited spatial resolution. Recently, the location of RNAPII has been more precisely mapped relative to the nascent transcript, rather than the DNA template, using nascent transcript sequencing (NET-seq) or UV crosslinking and analysis of cDNA (CRAC) (Churchman and Weissman 2012; Holmes et al. 2015; Mayer et al. 2015; Nojima et al. 2015; Milligan et al. 2016).

To better characterize overall RNAPIII transcription in yeast, we employed CRAC using HTP—tagged form of the largest, catalytic subunit of RNAPIII (Rpo31, commonly known as Rpc160). High sequence coverage allowed accurate mapping of the location of RNAPIII along all nascent transcripts.

## Results

**Figure 1.**
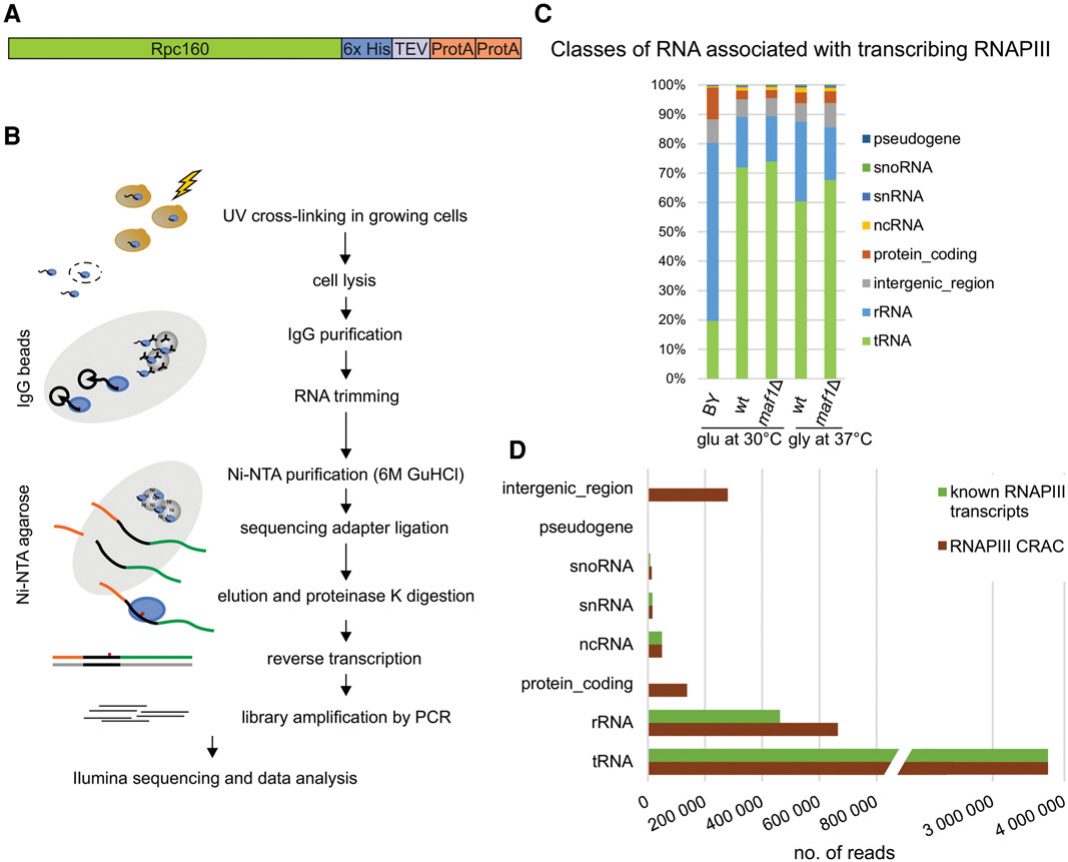
Outline and validation of RNAPIII CRAC. (*A*) Scheme of Rpo31-HTP fusion (commonly known as Rpc160-HTP) including a C-terminal His6-TEV protease cleavage site-protein A (HTP) tag for CRAC purification. (*B*) Outline of the CRAC crosslinking protocol. (*C*) Transcriptome-wide binding profiles for *MAF1* (wt) and *maf1*Δ strains encoding Rpo31-HTP and for the control BY strain expressing untagged Rpo31. Bar diagrams illustrate the percentage of all sequences mapped to each of the RNA classes indicated on the *right* of the figure. (*D*) Graphical comparison of the total numbers of reads recovered in RNAPIII CRAC with reads from known RNAPIII transcripts, divided into classes.

To permit the application of CRAC to RNAPIII, Rpo31 encoding the RNAPIII catalytic subunit was expressed as a fusion with a tripartite tag (His_6_–TEV protease cleavage site–protein A [HTP]). The Rpo31-HTP fusion protein (Fig. 1A) was expressed under the control of the endogenous *RPO31* promoter from the chromosomal locus and was the only source of Rpo31 in the cell. Strains expressing Rpo31-HTP showed wild-type (wt) growth rates, demonstrating that the fusion protein is functional (Supplemental Fig. S1A,B).

To crosslink Rpo31-HTP to the RNA, cells actively growing in glucose medium at 30°C or glycerol medium at 37°C were UV irradiated at 254 nm for ∼100 sec. As a negative control, the nontagged, parental strain BY4741 (BY) was used. The CRAC analysis was performed as previously described (for overview, see Fig. 1B; Granneman et al. 2009, 2010). Briefly, following cell lysis, protein–RNA complexes were isolated by immunopurification and affinity purification. RNA was partially digested with RNase A + T1; linkers were ligated to the 5′ and 3′ termini; protein–RNA complexes were recovered by SDS-PAGE (Supplemental Fig. S1C); and a cDNA library was prepared.

The sequencing generated 50-bp reads, and to ensure that bona fide 3′-ends were mapped, we analyzed only reads that included both the 5′ and 3′ linkers. Sequences were mapped to the yeast genome using NovoAlign (Novocraft). Data analysis is further described in the Methods and Supplemental Figure S2. Hits were counted using pyCRAC software (Webb et al. 2014) using a modified genome features file (GTF) as described in the Methods. As expected, tRNA genes were highly represented in each of the Rpo31-HTP data sets, relative to the BY control strain, in both the wt and *maf1*Δ backgrounds (Fig. 1C; Supplemental Table S1A). Transfer to glycerol medium at 37°C reduced the relative recovery of tRNA reads and increased rRNA reads (predominantly 5S rRNA) (Fig. 1C; Supplemental Table S1B) in the wt strain but not in *maf1*Δ, consistent with Maf1-mediated repression of tRNA transcription.

Analysis of the hit distribution confirmed the association of RNAPIII with nuclear-encoded tRNA genes (Fig. 1D), since mitochondrial tRNA genes represented only 0.001% of all tRNA reads (Supplemental Table S1C). A low recovery of sequences annotated as protein-coding was observed, but further analysis revealed that these predominantly represent regions that are in close proximity to tRNA genes. Known RNAPIII transcripts were also predominant among other small RNAs recovered (Fig. 1D). As an example, Supplemental Table S1C shows that U6 snRNA (encoded by the RNAPIII-transcribed gene *SNR6*) was much more frequently recovered than U1–U5 snRNAs, which are RNAPII transcripts. Similarly, the 5S rRNA (encoded by *RDN5*) represented 70% of all annotated rRNA sequences (Fig. 1D; Supplemental Table S1B).

Together these analyses confirm that the Rpo31 CRAC predominantly recovered authentic RNAPIII transcripts.

### Genome-wide analysis of tRNA transcription

**Figure 2.**
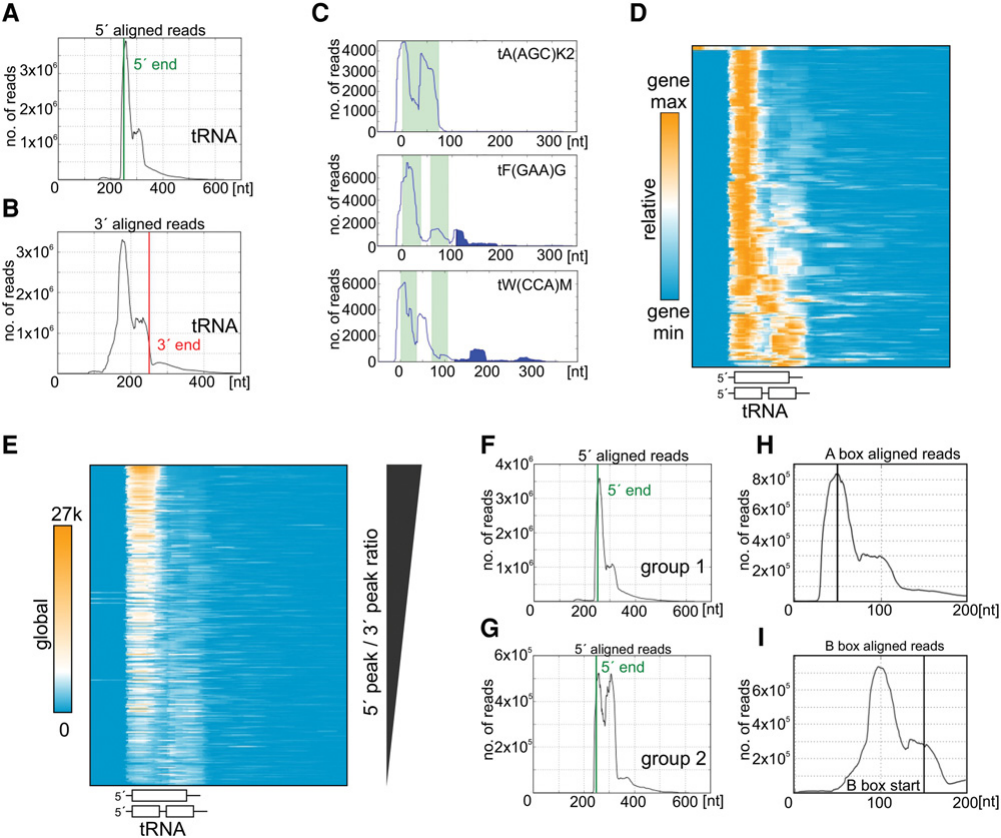
Unequal distribution of RNAPIII along tRNA genes indicated by genome-wide analysis. (*A*) Cumulative plot of all nuclear tRNA genes aligned to the mature 5′-ends. Distance (nt) is shown starting 200 nt upstream of the mature 5′-ends (designated by vertical green line) on the *x*-axis. (*B*) Cumulative plot aligned to 3′-ends of mature tRNAs (designated by vertical red line). (*C*) Hit distribution along individual genes [tA(AGC)K2, tF(GAA)G, tW(CCA)M], showing uneven patterns of RNAPIII occupancy. The *x*-axis shows distance (nt) from the 5′-end of the mature tRNA. The light green background indicates the exonic region(s). The area filled in blue represents RNAPIII read-through of the canonical termination signal. Plots for all tRNA genes are presented in Supplemental Data Set D1. (*D*) Heatmap for all nuclear tRNA genes with 50-nt 5′ flanks and 150-nt 3′ flanks, clustered using the 1-Pearson coefficient. The hit intensity scale is shown relative to the maximum value within each gene. The 5′ peak is the predominant feature. (*E*) All nuclear tRNA genes were ordered according to 5′ to 3′ peak ratios. The color intensity compares the number of reads to the global distribution of mapped reads. (*F*) Genome-wide profile of 225 tRNA genes aligned to 5′-ends of mature tRNAs. These genes (group 1) show the predominant pattern with a higher 5′ peak (5′ to 3′ peak ratio >1.5). (*G*) Genome-wide profile of the remaining 50 tRNA genes (group 2) showing the less common pattern with similar-sized 5′ and 3′ peaks (5′ to 3′ peak ratio <1.5). (*H*) Genome-wide profile of all nuclear tRNA genes aligned to A box. The start of the A box sequence is indicated by a vertical line. (*I*) Profile aligned to B box. The start of the B box sequence is indicated by vertical line.

The density of RNAPIII was compared across all individual tRNA genes. Raw reads were analyzed. Figure 2, A and B, shows cumulative plots of primary transcripts aligned by the 5′-end or 3′-end of the mature tRNA, for yeast grown on glucose medium at 30°C. Apparent transcription read-through (RT) was observed on many tRNA genes. This is indicated by RNAPIII density beyond the canonical termination site located just 3′ to the end of the mature tRNA (Fig. 2B, red line). Broadly similar distributions of RNAPIII density reads were observed during growth in glycerol medium at 37°C and in the absence of Maf1 (Supplemental Fig. S3).

Inspection of individual tRNA transcription units revealed notably uneven RNAPIII density across the mature tRNA and flanking pre-tRNA regions, as well as over 3′-extensions when present. Representative, individual tRNAs are presented in Figure 2C; other RNAPIII transcription units are shown in Supplemental Figure S4. Graphs showing the distribution of RNAPIII across all individual tRNA genes are provided in Supplemental Data Set D1. Figure 2D shows a heatmap displaying the distribution of reads across each tRNA gene, relative to the maximum for the gene. This revealed a predominant pattern across most genes, with a high density of reads over the 5′-end of the transcription unit and a weaker peak before the 3′-end of the mature tRNA. A minority of genes showed similar 5′ and 3′ peaks.

For each gene, the ratio between the numbers of reads corresponding to the 5′ and 3′ peaks was determined. This was plotted relative to total number of reads mapped to that gene, as an indication of transcription rate (Supplemental Fig. S5A). This revealed that the 5′ “initiation” peak is relatively larger for highly transcribed genes. Measuring the areas under the 5′ and 3′ peaks confirmed that a higher transcription rate correlates with higher ratio of 5′ to 3′ peaks (Supplemental Fig. S5B). Ordering genes according to their 5′ to 3′ peak ratio on global heatmap clearly shows that the 5′ peak is predominant in highly transcribed genes (Fig. 2E). However, under conditions of reduced RNAPIII transcription (glycerol medium at 37°C), the correlation between the 5′ to 3′ peak ratio and transcription rate was greatly reduced (Supplemental Fig. S5C–E).

Clustering using 5′ to 3′ peak ratio revealed two major patterns. Group 1 showed a very high 5′ peak with a much lower 3′ peak and included 225 of 275 genes, representing all 20 isotypes and 40 isoacceptors (Fig. 2F). Group 2 showed similar-sized 5′ and 3′ peaks and was less common, including 50 of 275 genes and representing 14 isotypes and 16 isoacceptors (Fig. 2G). Moreover, tRNAs genes falling into group 2 showed higher overall levels of RNAPIII transcription termination RT (see below).

On tRNA genes, the spacing between the 5′ and 3′ peaks was ∼50 nt. The approximate footprint expected for the RNAPIII complex is 38 nt (Hoffmann et al. 2015), suggesting that the observed peaks might reflect pileups caused by the high RNAPIII density. However, a consistent pattern of ∼50 nt spacing across all tRNA genes was not obvious (Supplemental Fig. S6A). Rather, the 5′ and 3′ peaks, indicating a high density of RNAPIII, appear to be localized with respect to the A and B boxes of the internal promoter. Two examples of tRNA genes are presented in Supplemental Fig. S6B, with the A and B boxes marked. Genome-wide profiles of RNAPIII density aligned to the A or B boxes revealed that the initiation-associated 5′ peak is located at the beginning of the A box (Fig. 2H); the 3′ peak is located at the beginning of the B box (Fig. 2I). The same was true for the group 2 tRNAs (50 genes with 5′ to 3′ peaks ratio <1.5) (Supplemental Fig. S6C, panels I and II) and for intron-containing genes, in which the relative position of box B to box A is variable since they are separated by the intronic sequence (Supplemental Fig. S6C, panels III and IV). We postulate that TFIIIC bound to the A and B boxes can interact with and impede transcribing RNAPIII, leading to transient pausing.

### Novel RNAPIII transcripts

**Figure 3.**
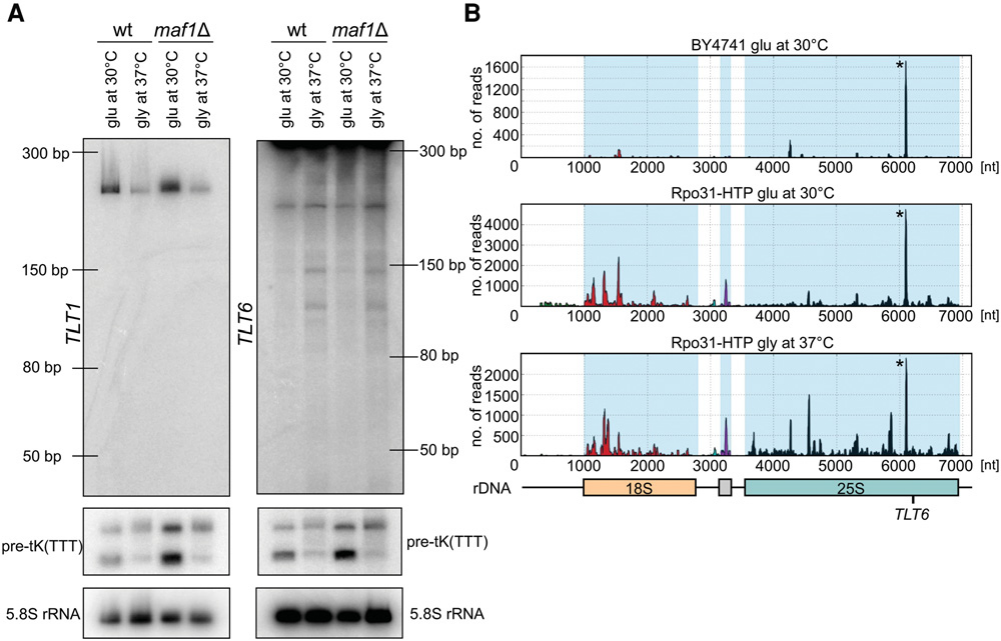
RNAPIII CRAC reveals new RNAPIII transcripts. (*A*) Putative new RNAPIII transcripts were confirmed by Northern blotting using specific probes against *TLT1* (oTWT002) or *TLT6* (oTWT007) and the 5.8S rRNA loading control. (*B*) The Rpo31 (RNAPIII) distribution over the *RDN37* gene encoding ribosomal rRNA reveals patterns characteristic of RNAPIII transcription units with the 18S region (red). A common contaminant peak seen in many CRAC analyses is marked with asterisk.

In addition to the protein coding genes and well-defined stable RNAs, yeast RNAPII transcribes very large numbers of noncoding RNAs, largely of unknown function, and also generates a low level of transcription throughout the majority of the genome (see Porrua and Libri 2015 and references therein). In contrast, yeast RNAPIII appears to be restricted to the expression of well-defined genes, with accurate recognition of transcription start sites. We were, however, able to identify a number of previously unrecognized, putative RNAPIII transcripts. By focusing on nonannotated regions of the genome (for details of regions selected, see Methods), we found six potential new RNAPIII genes designated tRNA-like transcripts 1 to 6 (*TLT1-6*) (Supplemental Table S2; Supplemental GTF File). In initial analyses, the expression of *TLT1* and *TLT6* was confirmed by Northern hybridization (Fig. 3A), and each is expressed at relatively high levels. Notably, *TLT6* is closely homologous to an apparent fragment of the 37S rDNA gene (*RDN37*) that is not located in the rDNA locus. In addition, we identified distinct Rpo31-HTP–associated transcripts that were located within the RNAPI-transcribed *RDN37* rDNA, particularly at the 5′-end of the 18S rRNA region (Fig. 3B, middle and bottom panel). These showed the characteristic double-peak profile of RNAPIII distribution, as observed over tRNA genes, and were absent from the BY4741 control (Fig. 3B, upper panel). The prominent peaks visible in the 3′ region of 25S rRNA (marked with asterisk) in all data sets represent a common contaminant seen in many CRAC analyses (Granneman et al. 2010; Lebaron et al. 2013; Tuck and Tollervey 2013; Turowski et al. 2014). We confirmed the presence of small RNAs derived from the rDNA gene, which were more abundant during growth on glycerol at 37°C (Supplemental Fig. S7). We speculate that RNAPIII transcription units may be active in the accessible rDNA repeats that are not transcribed by RNAPI.

### RNAPIII transcription levels under different growth conditions

**Figure 4.**
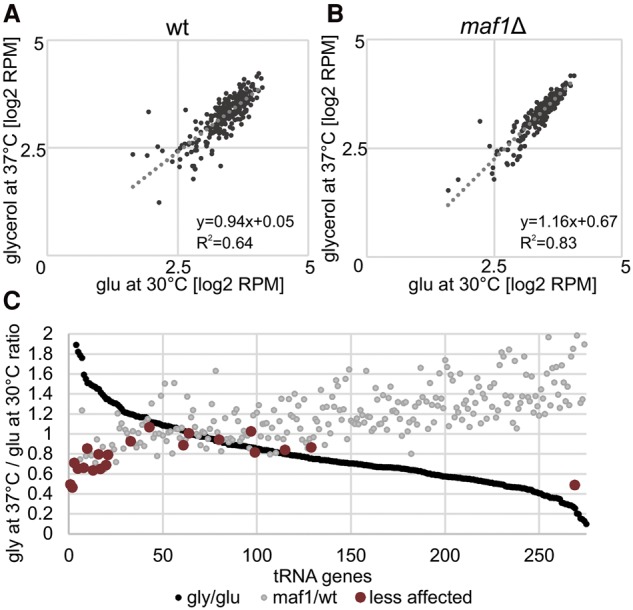
Comparison of genome-wide tRNA genes transcription under different growth conditions. (*A*) Correlation of relative expression of nuclear tRNA genes between permissive and repressive conditions for wt strain. The correlation coefficient (*R*^2^) reflects how well the data fit the trend line. Two outlying bottom points are not shown. (*B*) Correlation of relative expression of nuclear tRNA genes between permissive (glucose at 30°C) and repressive conditions (glycerol at 37°C) for *maf1*Δ strain. Two outlying bottom points are not shown. (*C*) Ratios of relative expression level of all nuclear tRNA genes under stress conditions. A subset of tRNA genes is less responsive to regulation by Maf1. Genes are ordered according to the Gly:Glu expression ratio (the highest value for tD(GUC)N = 14.78 is not shown). In the same order, the *maf1*Δ:wt ratio is plotted. The least affected tRNA for each amino acid is colored in dark red.

We compared relative transcription by RNAPIII over all nuclear tRNA genes under near optimal growth conditions (glucose medium at 30°C) and following transfer to stress conditions known to repress tRNA expression (glycerol medium at 37°C). To allow comparison of genes with identical mature regions, specifically for this analysis we excluded all reads mapping to more than one site in the genome. This eliminates tRNA fragments that would be ambiguously mapped to the mature tRNA regions of more than one gene (Supplemental Fig. S2C). We were able to compare expression levels using gene-specific reads that overlap the mature tRNA ends and the gene-specific, flanking regions. Under stress conditions, reduced transcription was observed for nearly all tRNAs, as previously reported (Ciesla et al. 2007), but the degree of repression was highly variable between tRNA genes (Fig. 4A). Some tRNA genes appeared to have elevated RNAPIII occupancy (Gly:Glu ratios >1.0). This apparent increase is probably a consequence of normalization to total RNAPIII reads under conditions in which most tRNA genes are strongly repressed, but clearly shows that transcription of a subset of tRNA genes is less repressed following glucose withdrawal. This conclusion is broadly consistent with a previous microarray analysis, which revealed that the levels of mature tRNAs are reduced to variable extents on a nonfermentable carbon source (Ciesla et al. 2007).

Maf1 is an RNAPIII repressor that acts following nutritional downshift, particularly at elevated temperatures (Ciesla et al. 2007; Boguta 2013). To assess the role of Maf1 in repressing transcription of each tRNA gene, we plotted the ratio of RNAPIII occupancy following transfer between permissive (glucose at 30°C) and restrictive (glycerol at 37°C) conditions for wt (Fig. 4A) and *maf1*Δ data sets (Fig. 4B). Decreased repression in the absence of Maf1 is shown by the increased slope of the trend line in *maf1*Δ (*y* = 1.16*x* − 0.67) relative to the wt (*y* = 0.94*x* + 0.05).

In addition, it is evident that the heterogeneity in tRNA repression seen in the wt is substantially reduced in the absence of Maf1. This was confirmed by the increased correlation coefficient (*R*^2^) in *maf1*Δ (*R*^2^ = 0.83) relative to the wt (*R*^2^ = 0.64). This provides genome-wide evidence that Maf1 does not simply down-regulate all tRNAs but provides an additional layer of gene-specific RNAPIII regulation.

Figure 4C shows all tRNA genes ranked by the ratio of expression in glucose at 30°C versus glycerol at 37°C (the string of black points which form the line in Fig. 4C), with the ratio of expression in *maf1*Δ versus *MAF1* shown in gray. In general, tRNA genes that show low repression under stress conditions also show low relative overexpression in *maf1*Δ strains (*maf1*Δ: wt ratio <1.0). This indicates that a subset of tRNA genes shows low responsiveness to both environmental and cellular signals. Notably, this group contains at least one tRNA for each amino acid. In Figure 4C, the least affected tRNA for each amino acid is indicated in dark red. The only exception was *SUP53*, encoding tL(CAA)C, which was very insensitive to Maf1 relative to most tRNAs but was strongly repressed by transfer to glycerol at 37°C, suggesting a distinct mode of regulation. Together these findings suggest the existence of a basal subset of “housekeeping” tRNA genes. This model is consistent with conclusions based on Maf1-mediated repression of actively transcribed tRNA genes in human cells subjected to serum starvation (Orioli et al. 2016).

Analysis of the RNAPIII profiles along all tRNA genes revealed very few differences between wt and *maf1*Δ strains (see Supplemental Fig. S3; Supplemental Data Set D1). This is in contrast to the changes in total RNAPIII density and strongly indicates that Maf1, which acts during RNAPIII transcription initiation (Desai et al. 2005; Vannini et al. 2010), does not interfere with RNAPIII elongation.

A subset of other RNAPIII transcripts was strongly increased under stress conditions (Supplemental Table S3). The most elevated was *RNA170*, transcription of which was up-regulated nearly 100-fold in a Maf1-independent manner. Surprisingly, numerous ncRNAs that are generally transcribed by RNAPII showed apparent increases in RNAPIII transcription of greater than twofold under stress conditions. These included several small nucleolar RNAs (snoRNAs), notably *SNR189* and the *LSR1* (encoding U2 small nuclear RNA). It appears that under stress conditions, these RNAs are increasingly transcribed by RNAPIII. This could reflect a loss of RNAPII transcription, increased availability of RNAPIII due to decreased tRNA expression, or a combination of these changes.

Other known RNAPIII transcripts, including *SNR6*, *SCR1*, and *RPR1*, showed mild increases (1.4- to 1.9-fold) in apparent RNAPIII transcription following glucose withdrawal (Supplemental Table S3). These modest apparent changes may simply reflect decreased tRNA transcription, since the reads are relative to total RNAPIII binding.

### Termination RT on RNAPIII transcription units

**Figure 5.**
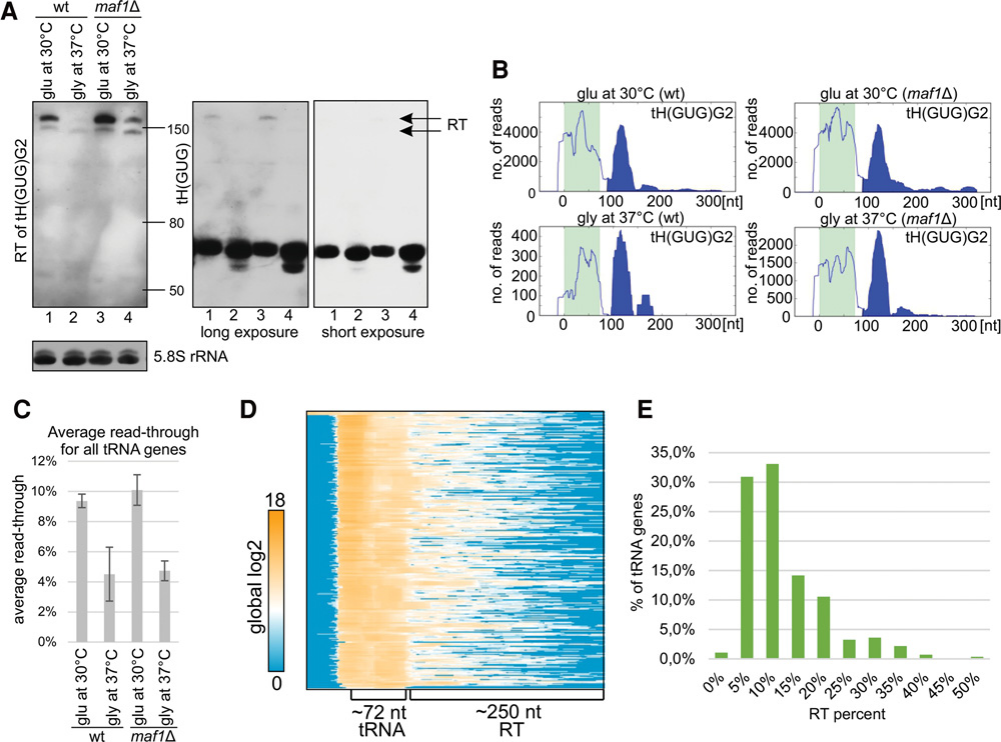
Transcription termination read-through (RT) by RNAPIII. (*A*) Northern blotting reveals extended transcripts from gene tH(GUG)G2 (*left*). Total RNA extracts were analyzed by Northern blotting using probes specific to the RT sequence 54 nt downstream from the tRNA (*left*), within tH(GUG) (*right*), and 5.8S rRNA (loading control). Extended forms are of low abundance relative to mature tH(GUG). (*B*) Distribution of read density over gene tH(GUG)G2 under conditions presented in *A*. These CRAC results are consistent with the Northern blot but indicate the instability of extended transcripts. (*C*) Average RT levels for all nuclear tRNA genes. RT was calculated for each gene under indicated conditions separately, and average RT values for all nuclear tRNA genes are presented. (*D*) Heatmap for all nuclear tRNA genes, with 50-nt 5′ flanks and 250-nt 3′ flanks, clustered using the 1-Pearson coefficient. Hits intensity is presented as log2 of global values. Regions of mature tRNA genes and RT are labeled. (*E*) Histogram showing distribution of RT among all tRNA genes.

The major termination sites on yeast tRNA genes have long been identified by the presence of oligo(U) tracts in the transcript, located 5–15 nt downstream from the 3′-end of the mature tRNA sequence (Orioli et al. 2011). Perhaps the most unexpected finding from our analyses was the identification of substantial RT transcription on many tRNA genes, typically extending 50–200 nt beyond the expected terminator (Fig. 5). The RT distribution detected in vivo using CRAC was quite different from previous analyses of in vitro transcription (see Discussion) (Table 1; Braglia et al. 2005).

**Table 1.**
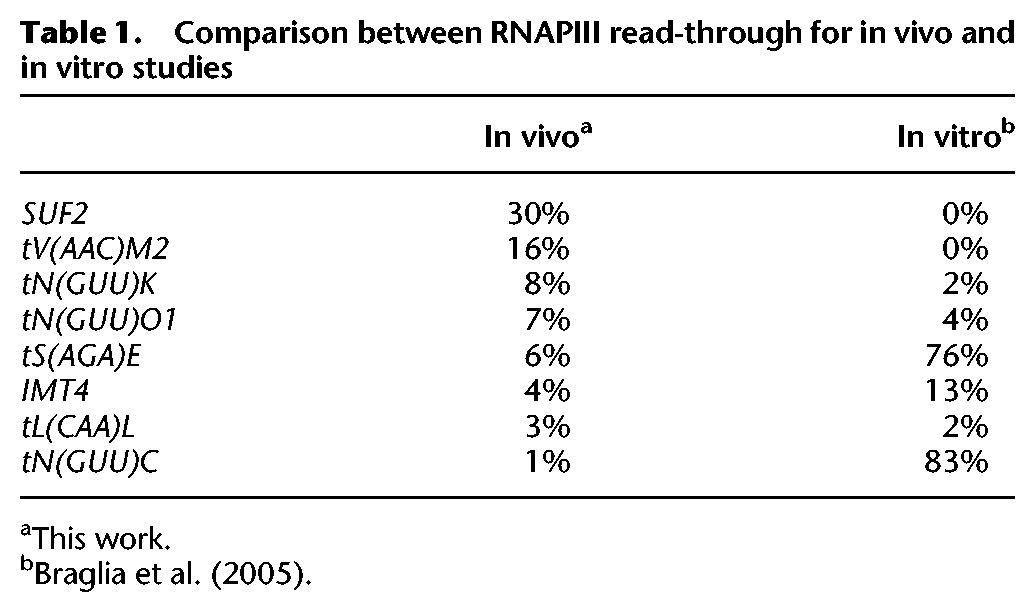
Comparison between RNAPIII read-through for in vivo and in vitro studies

To confirm the CRAC data and test whether RNAPIII generates the expected long, continuous transcripts, we performed Northern hybridization for tRNAs showing high RT levels (Fig. 5A; Supplemental Fig. S5A–C). Probes specific for mature tRNA and extensions were used to assess the presence of 3′ extended pre-tRNA transcripts and estimate ratios between mature and 3′ extended tRNAs. Clear RT bands were identified using tRNA-specific probes for tH(GUG) (Fig. 5A), tE(UUC), tK(UUU), and tY(GUA) (Supplemental Fig. S8A–C). The RT bands were generally less strong for yeast grown on glycerol medium at 37°C, presumably due to down-regulation of RNAPIII. RT products from the tH(GUG)G2 and tK(UUU)O genes were detected using a specific probe, against regions 54 nt 3′ to the mature tH(GUG)G2 (Fig. 5A, left) and 40 nt 3′ to the mature tK(UUU)O (Supplemental Fig. S8B). The abundance of the extended transcripts relative to mature tRNAs (Fig. 5A, right) was substantially lower than the degree of RT seen in the CRAC analyses (Fig. 5B), indicating they are rapidly processed or degraded (see below). The RT transcripts were not previously recognized as pre-tRNAs, probably due to the combination of their lengths and low abundance.

To identify features associated with RT efficiency, RT levels were calculated for each individual gene. This was defined by the ratio between the number of reads mapping to extensions (regions >15 nt 3′ to the mature tRNA) to total pre-tRNA reads (from −15 nt 5′ to the mature tRNA gene to the 3′-end of the RT product). Average RT levels for all tRNA genes were >10% in strains growing under permissive conditions (Fig. 5C). RT decreased following transfer to repressive conditions but was unaffected by the loss of Maf1, consistent with elongation being independent of Maf1. To visualize the overall frequency of transcriptional RT under permissive growth conditions, a global log2 heatmap is presented for all tRNA genes (Fig. 5D).

**Figure 6.**
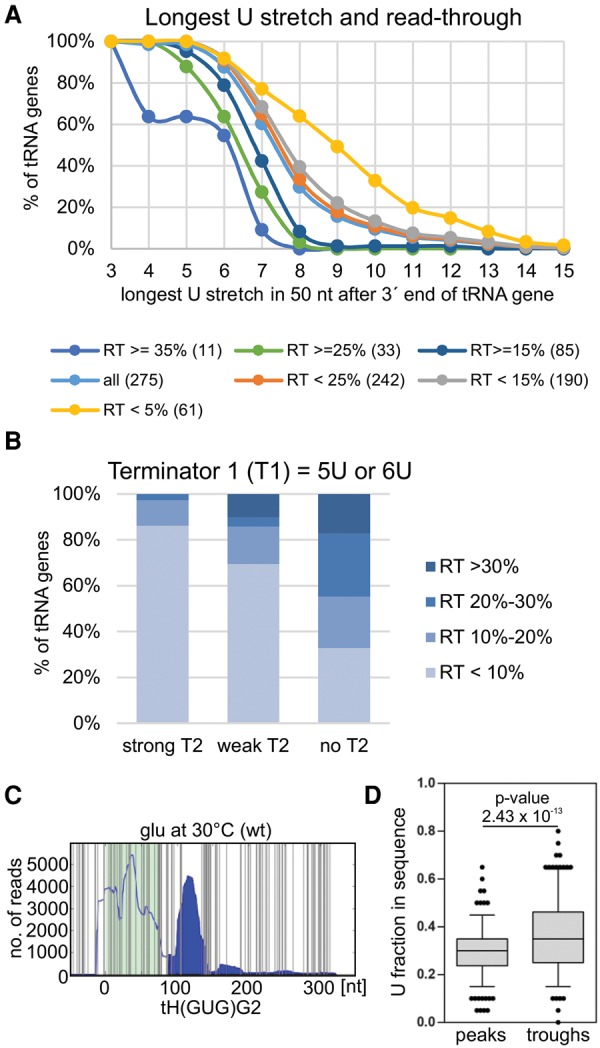
Canonical and noncanonical RNAPIII termination signals. (*A*) Read-though (RT) level depends on the strength of the canonical termination signal. Cumulative plot showing distribution of the longest U stretch within 50 nt downstream from the tRNA 3′-end. All tRNA genes were divided according to RT level (<5%, <15%, <25%, >15%, >25%, and >35%). Numbers of genes are presented in parentheses. (*B*) A weak first (canonical) terminator (T1) is often supported by a second terminator (T2). Analysis of secondary termination signal in a 40-nt range from first termination signal for weak terminators T1 = 5U and T1 = 6U. The second terminator is classified as strong (more than 6U), weak (less than 6U), or absent (no clear T2). (*C*) Uracil abundance is a noncanonical termination signal within read-through regions. Hit distributions along tH(GUG)G2 with all U nucleotides marked (vertical gray lines) revealed a correlation between U frequency and RNAPIII transcription/termination. Green background indicates the exon position(s). Filled area indicates RNAPIII read-through. (*D*) Boxplot and line plot showing the distribution of uracil abundance in peaks and following troughs within RT. The first, canonical termination sites were excluded from this analysis. *P*-value = 2.43 × 10^−13^ was calculated using Wilcoxon test for peaks with average reads above 300 and considering a 20-nt region comprising 10 nt before and 10 nt after each peak/trough.

Even genes with relatively high RT levels showed a sharp drop in RNAPIII density following the 3′-end of the mature tRNA (Fig. 2B; Supplemental Fig. S8D), indicating that transcription termination generally occurs at canonical termination signals. However, the range of RT levels (Fig. 5E; Supplemental Fig. S8E) shows that the efficiency of canonical termination is highly variable. To identify features associated with efficient termination, we compared genes showing different levels of RT (Fig. 6A; Supplemental Fig. S8F). As expected, RT levels were negatively associated with the length of the oligo(U) tract at the canonical termination site. Genes showing higher RT have a clear tendency to have weaker canonical termination signals over the 50 nt 3′ to the mature tRNA sequence (Fig. 6A). This also indicated that effective canonical termination of RNAPIII in vivo needs terminators stronger than a 6U tract. Moreover, for genes with >25% RT, >60% have 6U tracts as the longest termination signal, whereas for genes with <5% RT, 60% have 8U tracts. The most common poly(U) tracts within 50 nt 3′ to the mature sequence across all tRNA gene terminators are 7U and 8U. Analysis of 3′-extensions revealed that genes with high RT levels also lack a second canonical termination signal within the next 40 nt (Fig. 6B).

Within the 3′-extensions, a clear negative correlation was seen between RNAPIII density and the frequency of U residues. Figure 6C and Supplemental Figure S6A present hit distributions across RT regions with the U residues indicated. A low frequency of U residues was correlated with accumulation of RNAPIII, even 100–150 nt downstream from the canonical termination site. We confirmed these findings by statistical analysis of U enrichment within peaks and troughs in the RNAPIII density in the RT region. The canonical terminator was excluded from this analysis to avoid biasing the outcome. The results were highly significant (see legend to Fig. 6D). A box-and-whisker plot (Fig. 6D) clearly shows that U residues are more abundant within regions showing RNAPIII density minima (troughs). This was unexpected because oligo(U) tracts were reported to be associated with transcription pausing by RNAPIII (for review, see Arimbasseri et al. 2013). We speculate that on encountering a U-rich region, the nascent transcript may be rapidly lost from RNAPIII, perhaps due to the low stability of oligo(dA:rU) regions. However, the polymerase may remain transiently bound to the DNA, perhaps stabilized by the interactions with the dT sequence of the nontemplate strand (Arimbasseri and Maraia 2015). Since CRAC relies on RNA crosslinking, these polymerases would not be detected, giving rise to the observed RNAPIII density minima. Additionally, RNAPIII bound at these sites might transiently block elongation by subsequent polymerase molecules, potentially generating the upstream peaks in density.

The greatest RT was seen for a subset of tRNA isotypes (Supplemental Fig. S9B); RT >30% was observed for 20 genes representing 10 isotypes (Supplemental Fig. S8D). However, RT >15% was seen for 85 genes representing all isotypes, indicating only limited isotype dependency.

Formation of a RNA:DNA duplex (R-loop) facilitates termination by RNAPII (Komissarova et al. 2002; Skourti-Stathaki et al. 2014), suggesting that this might also be the case for RNAPIII. We therefore assessed the propensity for R-loop formation over the 20 nt downstream from each mature tRNA by comparison of the predicted ΔG for DNA/DNA and RNA/DNA duplexes (Supplemental Fig. S9C). Across all tRNA genes, no significant correlation between RT and ΔG was detected (Supplemental Fig. S9C, left). However, a weak tendency toward R-loop formation was observed for isotypes with the highest RT (RT > 25%) (Supplemental Fig S6C, right) relative to other genes (Supplemental Fig S9C, middle). We further sought to identify structural features that potentially facilitate termination. In silico analysis using RNA folding software failed to identify consistent correlations between the predicted presence or stability of structural elements in the nascent transcript and RT efficiency.

Altogether, oligo(U) tract length and uracil frequency were the only features in tRNA 3′ flanking regions that were consistently correlated with transcription RT levels in vivo.

### Surveillance of 3′ pre-tRNA extensions

**Figure 7.**
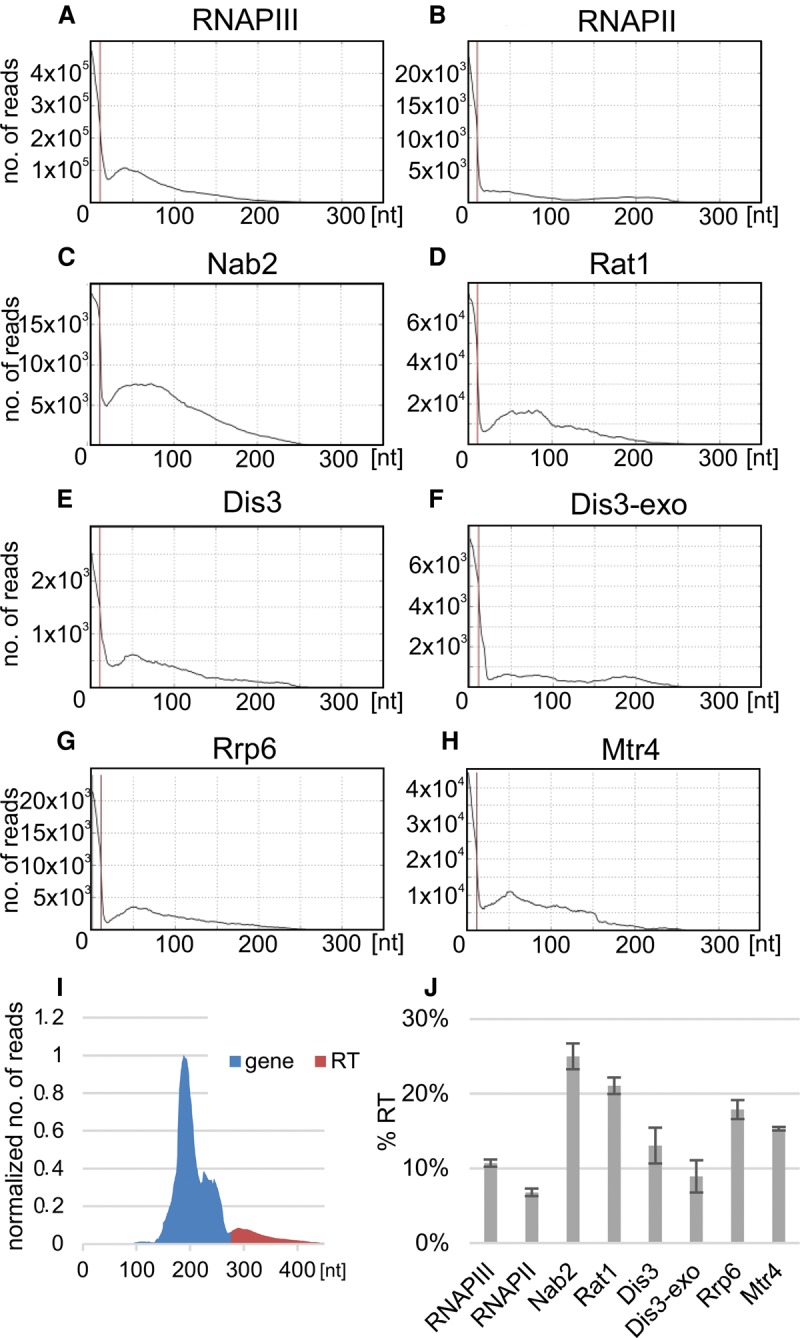
Genome-wide analysis of surveillance factors and its role in targeting transcriptional read-through of tRNA genes. Genome-wide profile of nuclear tRNA transcripts aligned to mature 3′-ends. Genome-wide hit distributions are shown for (*A*) RNAPIII (Rpo31), (*B*) RNAPII (Rpo21) (Holmes et al. 2015), (*C*) Nab2, (*D*) Rat1 (Granneman et al. 2011), (*E*) Dis3, (*F*) Dis3-exo, (*G*) Rrp6, and (*H*) Mtr4. (*I*) Schematic representation of genome-wide profile divided into gene and RT fragments. The frequency of binding to RT regions was calculated as RT fraction divided by total number of reads (gene plus RT). (*J*) Histogram showing frequency of binding to RT regions (in percentages) relative to total tRNA for genome-wide profiles.

The extended pre-tRNA species were not previously reported from RNA expression analyses in *S. cerevisiae*, and data in Figure 5A and Supplemental Fig. S5A–C also indicate their low steady-state levels, suggesting that they may be relatively unstable. Pre-tRNAs represent major targets for the nuclear surveillance machinery (Wlotzka et al. 2011; Gudipati et al. 2012; Schneider et al. 2012). To determine whether these 3′-extended RNAs are specifically targeted by the nuclear surveillance system, we analyzed CRAC data for known surveillance factors: Nab2, Mtr4, Rrp6, Dis3, and Rat1 (Fig. 7). To compare binding profiles, full-length tRNA transcription units were annotated using the RNAPIII CRAC data (Fig. 7A) to limit background from RNAPII transcription, which is present at low levels throughout the genome (Fig. 7B; Holmes et al. 2015; Milligan et al. 2016).

The RNA binding protein Nab2 is involved in surveillance (Schmid et al. 2012) and RNAPIII transcription (Reuter et al. 2015). Nab2 interacts directly with RNAPIII and TFIIIB at the 5′-end of tRNA genes (Reuter et al. 2015) and may couple transcription initiation and surveillance. In our analysis, Nab2 binding to tRNA 3′-extension was clearly elevated (Fig. 7C). Binding to 3′ extended pre-tRNAs was also observed for Rat1 (Fig. 7D), the major 5′-exonuclease for tRNA surveillance (Chernyakov et al. 2008), and for Dis3 (Fig. 7E), the major exosome-associated nuclease with 3′-exonuclease and endonuclease activities. Greatly reduced binding was seen for a Dis3 mutant lacking exonuclease activity (Dis3-exo) (Fig. 7F), and lower binding was also seen for the other exosome-associated nuclease Rrp6 (Fig. 7G). The major nuclear cofactor for the exosome is the RNA helicase Mtr4, which was also bound to tRNA 3′-extensions (Fig. 7H). The relative association of different factors with tRNA 3′-extensions was determined by comparing binding to the RT regions with total tRNA, in genome-wide profiles (Fig. 7I,J).

We conclude that 3′-extended pre-tRNAs are bound by the nuclear RNA surveillance machinery, potentially explaining their low steady-state levels.

## Discussion

Here we report the analysis of localization of actively transcribing RNAPIII by UV crosslinking to the nascent transcripts. A similar approach was recently applied to characterize RNAPII transcription (Holmes et al. 2015; Milligan et al. 2016). The data analysis applied here was similar to reported NET—seq analyses for RNAPII (Mayer et al. 2015; Nojima et al. 2015), with selection for short reads containing native 3′-ends. The RNAPIII binding sites identified were reproducible between experiments and included all previously reported RNAPIII transcription units. In addition, we identified novel RNAPIII transcription units, including transcripts generated from within the rDNA repeats, two of which were verified by Northern hybridization.

The CRAC analysis revealed notably uneven distribution of RNAPIII across transcription units. Pre-tRNA genes predominantly showed a strong 5′ peak and a weaker peak close to the 3′-end of the mature tRNA. A similar strong 5′ peak for tRNA genes was observed in recent work using biotin-based genomic run-on (BioGRO) (Jordan-Pla et al. 2015). The BioGRO technique is quite different from CRAC and does not involve PCR amplification step, so the similar conclusions support the reliability of tRNA metaprofiles presented here. The 3′ peak was also observed in BioGRO, but only in the longer intron-containing tRNA genes (Jordan-Pla et al. 2015), probably due to lower spatial resolution relative to CRAC. We interpret these RNAPIII peaks as representing regions where RNA binding is favored because the polymerase shows a decreased elongation rate and/or transient pausing. This could reflect many features, but comparison of peaks to the locations of the internal promoter elements suggests that transcription factors bound at these sites may contribute to decreased elongation. The 5′ peak of RNAPIII crosslinking was located at the beginning of the A box and the 3′ peak at the beginning of the B box (Fig. 2H,I). The transcription factor TFIIIC binds to both the A and B boxes (Acker et al. 2013), and in vitro studies indicate that TFIIIC–DNA interactions must be disrupted during RNAPIII elongation (Bardeleben et al. 1994). The complex of TFIIIB and TFIIIC on tRNA genes occupies a DNA length similar to that of nucleosomes, which are absent from tRNA genes (Nagarajavel et al. 2013). TFIIIC association with DNA increases during repression, and it has been proposed that in addition to its activity as transcription factor, TFIIIC occupies tRNA and other genes during transitions in RNAPIII activity (Arimbasseri et al. 2014b). We speculate that TFIIIC binding to the A and B boxes helps form a physical barrier in the chromatin that transiently interferes with RNAPIII elongation.

Following its recruitment by TFIIIB, RNAPIII becomes part of a closed preinitiation complex at the transcription start site (Vannini and Cramer 2012). Subsequent promoter melting and the initiation-to-elongation transition involve major conformational changes of RNAPIII, which likely enhance its processivity (Fernandez-Tornero et al. 2010). On highly transcribed genes, a prominent 5′ peak was observed close to the transcription initiation site (Fig. 2E), suggesting that initiation site clearance, i.e., dissociation from transcription factors, may be rate-limiting during RNAPIII transcription.

Analysis of changes in tRNA transcription identified a subset of genes that showed low response to either nutrient shift or loss of the transcription repressor Maf1. This group included tRNAs for each amino acid and potentially represents a basal subset of “housekeeping” tRNA genes. Notably, this conclusion is consistent with the profile of Maf1-mediated repression of actively transcribed tRNA genes in human cells subjected to serum starvation (Orioli et al. 2016). Recruitment of human RNAPIII was down-regulated at the majority of genes in a Maf1-dependent manner but was not significantly changed at 49 of 622 tRNA loci, which were therefore referred to as showing “stable” RNAPIII occupancy following serum starvation. As in yeast, the “housekeeping” human genes included representatives of most tRNA isotypes. Under stress conditions, we also observed increased RNAPIII transcription on a number of RNAPII genes encoding small ncRNAs. It is conceivable that RNAPIII provides a “backup” expression system under conditions that impair RNAPII activity.

RT of the canonical termination sites on tRNA genes emerged as a surprisingly prevalent feature of RNAPIII transcription. The resulting 3′-extensions were confirmed by Northern hybridization. Previous studies showed that human RNAPIII occupies regions downstream from the 3′-ends of many tRNA genes (Canella et al. 2010; Orioli et al. 2011). Analyses of the human genome further suggested the presence of terminators located ≥50 nt from the 3′-ends of tRNA coding sequences, in addition to terminator RT (Orioli et al. 2011). In *Schizosaccharomyces pombe* strains lacking the mediator complex subunit Srb2 (commonly known as Med20), RT tRNA transcripts are adenylated and targeted for degradation by the exosome (Carlsten et al. 2015). It seems likely that RT of canonical termination sites is a feature of RNAPIII in many systems.

A run of six U residues in the nascent transcript was previously defined as the termination signal in yeast RNAPIII genes (for review, see Arimbasseri et al. 2013). However, our analysis revealed that in vivo termination is significantly more effective with 7U–8U (Fig. 6A). We also sought to identify features that correlated with downstream termination sites on RT transcripts, including sequence context, predicted secondary structure, and spacing between short terminators. The most consistent correlation with transcription termination was seen for the frequency of U residues (Fig. 6D). Notably, uracil abundance was significantly greater at sites identified as troughs in the RNAPIII density, suggesting that these also correlate with termination in the 3′-extended regions.

Recent work suggested that RNAPIII pauses and backtracks on termination signals, with termination facilitated by a structural element in the nascent transcript (Nielsen et al. 2013), whereas a subsequent study found termination to be independent of transcript terminator–proximal RNA structure (Arimbasseri et al. 2014a). In our analysis (Supplemental Fig. S9C), we found no clear evidence for effects of structural elements on RNAPIII termination.

**Figure 8.**
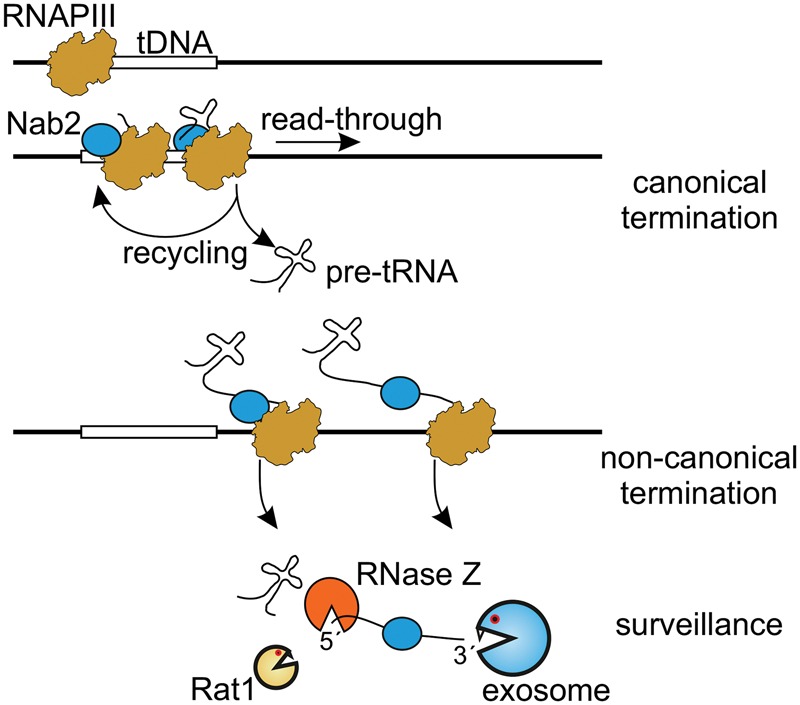
Model for noncanonical termination of RNAPIII. Nab2 accompanies RNAPIII on tDNA chromatin. When canonical termination occurs, the transcript is released and RNAPIII initiates another transcription cycle. When terminator read-through occurs, Nab2 targets the 3′ extended pre-tRNAs for degradation. Pre-tRNAs can be endonucleolytically cleaved by RNase Z (Trz1). Released tRNA extensions may be degraded by Rat1 and/or Mtr4 acting with the exosome or may be potentially processed to functional ncRNAs.

Extended tRNA transcripts likely escaped prior detection due to rapid turnover. This potentially involves recognition by the surveillance factor Nab2 and the TRAMP complex, followed by 5′ degradation by Rat1 and 3′ degradation by the exosome (Fig. 8). Nab2 is required for full RNAPIII transcription (Reuter et al. 2015), suggesting dual roles in transcription and surveillance. Binding of Nab2 to tRNA RT transcripts is also evident in published data (Tuck and Tollervey 2013; Reuter et al. 2015) but was not previously discussed. The exosome component Rrp6 has been implicated in the degradation of newly transcribed pre-tRNAs (Gudipati et al. 2012; Schneider et al. 2012). In contrast, stronger binding of the other exosome-associated nuclease Dis3 was seen on the RT transcripts. In Northern analyses, extension fragments gave clear band(s) consistent with the lengths of RT transcripts, indicating that they can be released through endonuclease cleavage. Pre-tRNAs can be 3′ processed by the endonucleases RNase Z (encoded by the *TRZ1* gene) (Takaku et al. 2003), and this cleavage might be followed by binding of Rat1 to the free 5′-end generated.

Previous analyses have implicated tRNA spacers and cleavage fragments as potential regulators of mRNA expression (Thompson and Parker 2009; Haussecker et al. 2010; Pederson 2010; Gebetsberger et al. 2012). Transcription RT appears to be a conserved feature, and we speculate that the products may also function in the regulation of gene expression.

## Methods

### In-vivo RNA crosslinking

CRAC experiments using Rpo31-HTP, Rpo31-HTP *maf1*Δ, Dis3-HTP, Dis3-exo-HTP, Rrp6-HTP, Mtr4-HTP, and Nab2-HTP were performed on cultures grown in SD medium with 2% glucose, lacking Trp to OD_600_ = 0.5. For nutrient shift, Rpo31-HTP and Rpo31-HTP *maf1*Δ strains were grown to OD_600_ = 0.5 in SD medium, harvested by filtration, transferred to medium containing 2% glycerol instead of glucose, and grown for an additional 2 h at 37°C. Actively growing cells were crosslinked in culture media (Granneman et al. 2011) and processed as previously described (Granneman et al. 2009, 2010). Total lysates were incubated with IgG-Sepharose for 2 h and extensively washed. Bound proteins were released by TEV cleavage. The eluate was RNase treated, and strong denaturing conditions were applied (6 M GuHCl). Tagged proteins were bound to Ni-NTA agarose, and the following steps were performed on the column: phosphatase treatment, 3′ linker ligation, RNA phosphorylation in the presence of [γ^32^P] ATP, and 5′ linker ligation. Tagged proteins were eluted, PAGE purified, and transferred to C+-Hybond membrane. RNA-associated proteins were identified by autoradiography, and bands of the correct size were excised and incubated with proteinase K to release bound RNA. Phenol-purified RNA was reverse transcribed and PCR amplified. Libraries were size-fractionated on agarose gels and sequenced using Illumina HiSeq with 50-bp single-end reads. To generate Nab2 data sets, all enzymatic steps were performed at 20°C.

### Data analysis

Data analysis is overviewed in Supplemental Figure S2A. Illumina sequencing data were collapsed to remove PCR duplicates. Rpo31-HTP and Rpo31-HTP *maf1*Δ data were preprocessed using FLEXBAR (Dodt et al. 2012) with the parameters –at 1 –ao 4 and were filtered to retain only reads containing the 3′ adaptor. Other data were preprocessed using FLEXBAR with default parameters. Data for Rat1 (Granneman et al. 2011) and RNAPII (Holmes et al. 2015) were previously published. During data processing, the raw sequence reads were “collapsed” by the removal of reads that are identical, including the random barcodes present in the linkers, since these potentially represent PCR duplicates. Across the mature tRNA regions, some fragments will be the same for multiple different genes that encode the same isoacceptor. It therefore seemed possible that collapsing the data might lead to the rejection of identical, but actually independent, reads with effects on global read distributions. To check this, we compared heatmaps for all tRNAs plotted using collapsed and uncollapsed data (Supplemental Fig. S2B). Because the patterns were the same, further analyses were performed using the smaller, collapsed data sets. All data sets were aligned to the yeast genome using NovoAlign (http://www.novocraft.com) with both –r random and –r none parameters as indicated in the text (Supplemental Fig. S2C).

All GTF annotation and genomic sequence files were obtained from Ensembl (*S. cerevisiae* genome version EF4.74) (Flicek et al. 2014). After initial analysis, genomic coordinates of tRNAs were arbitrary extended by 50 nt at the 5′-end and 250 nt at the 3′-end using BEDTools (Quinlan and Hall 2010). Experimentally confirmed 3′ tRNA extensions were annotated in a separate GTF file (Supplemental File 1). Potential new RNAPIII transcripts were annotated in separate GTF files (Supplemental File 2).

Downstream analysis was performed using pyCRAC software (Webb et al. 2014) and the gwide toolkit developed for this analysis (https://github.com/tturowski/gwide). PyReadCounters (pyCRAC) was used to calculate overlaps between aligned cDNAs and yeast genomic features, using a GTF file with extended tRNA genes. Single-gene and genome-wide plots were generated using the gwide toolkit. GENE-E was used to generate the heatmaps. Relative gene transcription was calculated as the number of reads from 15 nt upstream of the mature tRNA to the 3′-end of mature tRNA. 5′ to 3′ peak ratios were calculated dividing total read numbers from the 5′ half of each gene by reads in the 3′ half, where the genes were defined as extending from 15 nt upstream of mature tRNA to the 3′-end of mature tRNA. A and B boxes were identified using FIMO (Grant et al. 2011) from the MEME suite (Bailey et al. 2009) among all nuclear encoded tRNA genes.

tRNA RT was calculated from 15 nt to 250 nt downstream from the mature tRNA 3′-end. The longest U stretch in the 50 bp 3′ to the end of the tRNA was identified from FASTA sequences using simple unix command line tools: awk, grep, and wc. For identification of maxima and minima, the pypeaks package in the gwide toolkit was used (https://github.com/gopalkoduri/pypeaks). U-richness statistics using a Wilcoxon and paired *t*-test were calculated for various conditions using gwide toolkit: different average peak cut-offs, as well as 10-, 20-, and 50-nt regions around or before peaks and troughs. All *P*-values for 10 and 20 nt around peaks and troughs were significant (*P*-value <0.0002). Calculations for 50-nt surrounding sequences served as negative controls and resulted in *P*-values above 0.1.

New genes were identified using an sgr_peaks_identif.awk script (Supplemental File 3) and BEDTools (Quinlan and Hall 2010). Novofiles were converted into sgrs file (sgr format with added information about strand). All peaks at least 50 reads high and 10 nt long in *.sgrs files were identified using sgr_peaks_identif.awk and annotated. By use of BEDTools, annotated features were combined with merge –d 5 and intersected with all annotated overlapping genome features. New features were combined with merge –d 200 and annotated. All new features were manually analyzed: exclusion of overlaps with tRNA extensions, comparison with RNAPIII, RNAPII, and exosome data. Six new transcripts were annotated, and two were confirmed using Northern blots.

To compare data sets from different growth conditions, we excluded all reads mapping to more than one site in the genome. This eliminates tRNA fragments that would be ambiguously mapped to the mature tRNA regions of more than one gene. However, gene-specific reads overlapping the region between the TSS and the mature tRNA 5′-end, or between the 3′-end of the tRNA and terminator, will reflect transcription rates for individual tRNA genes. Data sets were normalized to hits per million mapped reads for analysis of RNAPIII transcription levels under different growth conditions. Other data are presented in raw read numbers since normalization would not change the shape of the profiles and spatial resolution, which are relevant in our study.

Yeast strains used in this study and the Supplemental Methods are available in the Supplemental Material.

## Data access

All RNA sequencing data from this study have been submitted to the NCBI Gene Expression Omnibus (GEO; http://www.ncbi.nlm.nih.gov/geo/) under accession number GSE77863.
